# The impact of a health promotion program on toddlers’ socio-emotional development: a cluster randomized study

**DOI:** 10.1186/s12889-024-17953-9

**Published:** 2024-02-09

**Authors:** Ana Duarte, Silvana Martins, Cláudia Augusto, Maria José Silva, Luís Lopes, Rute Santos, Rafaela Rosário

**Affiliations:** 1 School of Nursing, University of Minho, Campus de Gualtar, Braga, 4710-057 Portugal; 2 UICISA:E, The Health Sciences Research Unit: Nursing, School of Nursing of Coimbra, Coimbra, Portugal; 3 CIEnf, Research Centre in Nursing, School of Nursing, University of Minho, Braga, Portugal; 4 CIEC, Research Centre on Child Studies, Institute of Education, University of Minho, Braga, Portugal; 5 Research Centre in Physical Activity, Health and Leisure, Faculty of Sports, University of Porto, Porto, Portugal; 6 ProChild CoLAB Against Poverty and Social Exclusion – Association, Campus de Couros, Rua de Vila Flor, Guimarães, 4810-225 Portugal; 7 Institute of Education, University of Minho, Braga, Portugal

**Keywords:** Children, Child development, Health promotion, Socio-emotional skills, Community health

## Abstract

**Background:**

The first 3 years of life are a critical period for the development of socio-emotional skills, highlighting the importance of socio-emotional development in early childhood. This study aimed to evaluate the effectiveness of a health promotion intervention program on the socio-emotional development of children aged 12 to 42 months during the COVID-19 pandemic.

**Methods:**

A total of 344 children from 15 childcare centers participated in this study, with six centers in the intervention group and nine in the control group. Childcare teachers in the intervention group received a 6-month training program aimed at promoting healthy lifestyles, including topics such as diet, sleep, physical activity, and sedentary behavior. Sociodemographic and anthropometric measures were assessed at baseline, and socio-emotional development was assessed using the Bayley Scales of Infant and Toddler Development – Third Edition (Bayley-III) at baseline and post-intervention.

**Results:**

After the intervention, a significant difference in socio-emotional development was observed between children with mothers of varying education levels. Specifically, children whose mothers had lower education levels demonstrated significantly greater socio-emotional development (B = 19.000, *p* = 0.028) compared to the control group. In contrast, there was no significant difference in socio-emotional development among children with mothers from higher education levels.

**Conclusion:**

These findings suggest that intervention programs for childcare teachers can effectively promote healthy socio-emotional development in children from socioeconomically disadvantaged backgrounds. Future intervention programs should consider tailoring their approaches to target disadvantaged populations.

**Trial registration:**

This cluster randomized controlled trial was registered in the Clinical Trials database/platform on 09/09/2019 (number NCT04082247).

## Background

The bioecological theory is one of the theories that underlie child development. It states that humans develop through relationships between the child, family, community, programs, policies, systems, and the world at large [1]. Childhood development is the process through which children acquire cognitive, emotional, social, and physical skills during the early years of life [2]. This phase is considered critical for the development of various functions and skills, including emotional regulation, which is fundamental for maintaining a healthy psychological well-being [2]. This is particularly important during the first 3 years of life when rapid growth and development occur, and emotional skills play a crucial role [2, 3]. Socio-emotional development involves the progressive ability to interact with and learn from the social environment, regulate and communicate emotions, and develop relationships [4].

During early childhood, poorer emotional self-regulation has been found to be a predictor of obesity in later years, such as around 11 years of age [5, 6].

Emotional self-regulation is a crucial aspect of an individual’s ability to manage their emotional responses effectively. It encompasses the capacity for effortful control, which involves the ability to inhibit or activate behaviors in alignment with long-term goals, and the control of impulsivity, characterized by a heightened sensitivity to rewards and a reduced capacity for response inhibition [2, 5]. This multifaceted concept plays a vital role in regulating emotional reactions and adapting them to the demands of various situations [2, 5].

In addition to this, inadequate emotional development can lead to various other consequences, such as emotional eating or sleep disorders [2, 6]. Children who struggle with emotional regulation may find it difficult to cope with new situations, particularly in the event of conflicts [2, 3]. As a result, stress and negative emotions during childhood are essential public health concerns due to their potential implications for psychological and physiological problems [3]. A healthy lifestyle and a positive parenting style seem to protect socio-emotional development in children [2, 7]. Health Promotion Theory and Social Cognitive Theory, for instance, emphasize that promoting children’s health involves the active engagement of both families and communities to foster the adoption of healthy habits and lifestyles [8]. To the best of our knowledge, there are few health promotion programs that target toddlers at the childcare level. Several studies have concentrated on health promotion interventions for children ranging from kindergarten to high school ages [9–11]. However, there is limited research regarding interventions targeted at childcare centers and addressing development delays [12]. Moreover, such interventions often overlook emotional regulation [2]. Despite the limited number of intervention programs implemented in this field, those focusing on emotional regulation have generally been effective [13]. Interventions focused on childcare centers have the potential to be highly effective, given that children spend a significant portion of their time there and receive much of their education about healthy habits and lifestyles within that environment [6]. Many preventive interventions have been implemented in childcare centers, including increasing physical activity, reducing screen time, and providing healthier food options [14]. Some interventions are exclusively school-based, while others extend to families [15] or even the broader community [16]. Despite these efforts, there is still much to be discovered regarding best practices in this area, particularly when considering the role of socioeconomic status and parental education, given their significance in the study of childhood development [17, 18].The present study aims to analyze the effectiveness of an intervention program, based on health promotion, on socio-emotional development in children aged 12 to 42 months.

## Methods

### Participants and study design

This randomized controlled trial was conducted between 2018 and 2021 in fifteen Portuguese childcare centers. Childcare centers were selected by a statistician using a block size randomization of three, designed to be representative across geographic locations and childcare sizes. Childcare centers were invited to participate in the study. A minimum of 20 children was required as an inclusion criterion, and no additional inclusion criteria were applied to the childcare centers. Children were recruited into the study through childcare centers using a family-oriented process, where parents and children were treated as family units.

The only exclusion criterion was the presence of any disability that prevented children from being assessed. Initially, parental consent was obtained for the child’s participation, followed by securing the child’s assent. The child’s assent was obtained during the assessment, where each child was individually asked about their willingness to participate, and oral assent was obtained.

A total of 344 children were enrolled in the study, 168 males (48.8%) and 176 females (51.2%). The mean age at baseline was 23.6 (6.3) months and 31.3 (6.4) on follow-up.

**Fig. 1:**
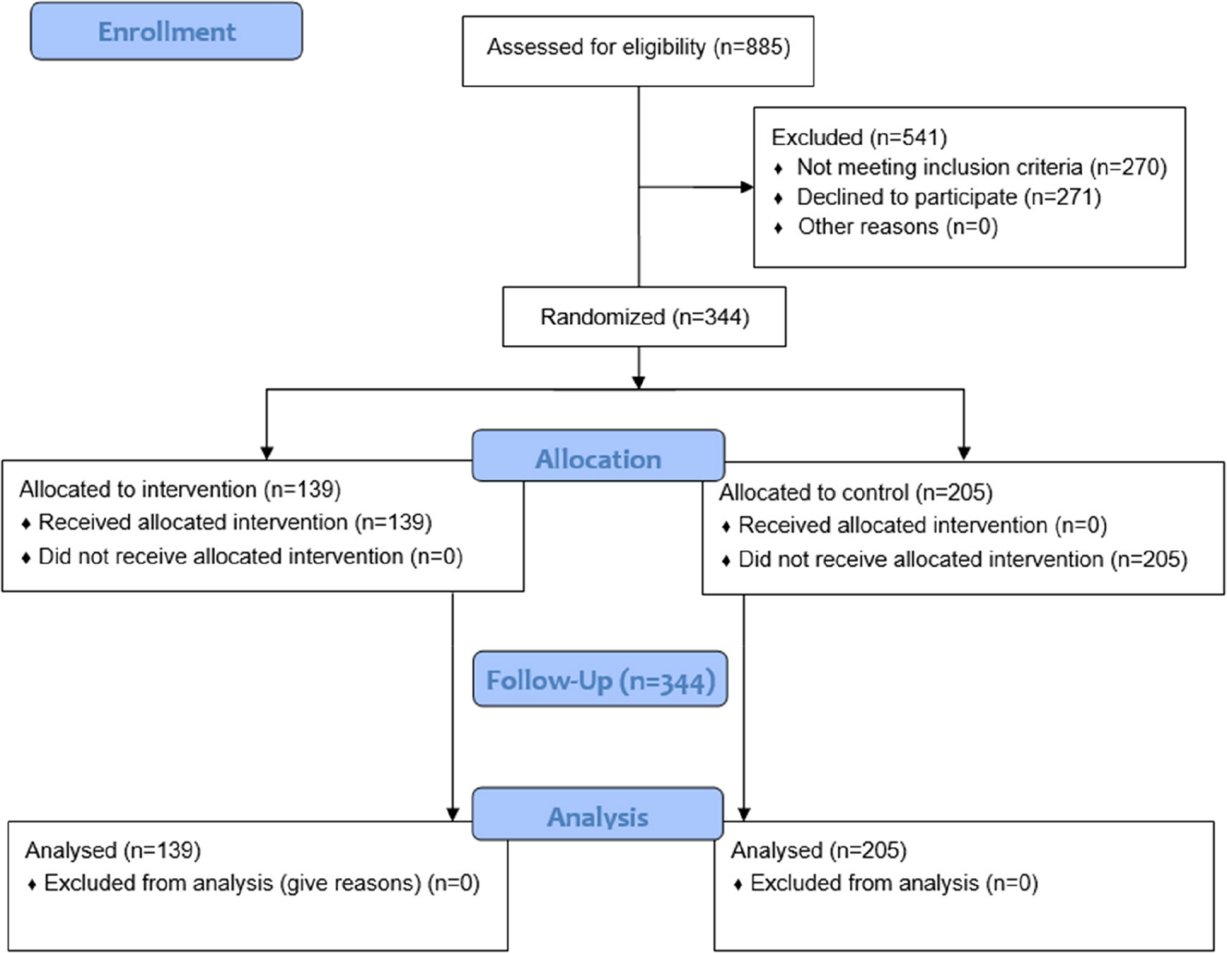
Flow diagram of participants through each stage of the project

To ensure allocation blinding of childcare centers, randomization was carried out after baseline data collection occurred (which occurred between October to December 2019). The random allocation was performed by an independent statistician. Due to the nature of both groups (intervention and control), clusters and participants were not blinded to their intervention allocation. Blinding was maintained for researchers who undertook follow-up assessments. Block randomization was performed and six childcare centers were allocated to the intervention group, which participated in the intervention program, while the other nine institutions belonged to the control group (Fig. 1 - diagram flow). In the randomization process, we considered variations in childcare center size, ranging from approximately 15 to 45 children. As a result, the control group ended up with a slightly higher number of clusters compared to the intervention group.

The control group didn’t receive any specific intervention during the implementation of the intervention program besides standard education and care. The follow-up assessments were performed between May and June 2020.

### Sociodemographic profile

Mother’s education level was assessed at baseline using a question extracted from the Graffar scale [19], adapted to Portugal. This is an international social classification, used as an indicator of the various welfare levels of a social group. It includes 5 criteria: occupation, level of education, sources of family income, housing comfort and appearance of the neighbourhood. Regarding mothers’ education, parents should indicate their last completed academic level. The answers were further transformed into less than higher education (low education) and higher education and more (high education). The assessment of the education level of mothers is a common practice in many studies and allows us to explore a significant variable that may influence child development [17, 20, 21]. Furthermore, an open-ended question was included to allow respondents to express the type of family structure to which they belonged.

### Anthropometric measures

At baseline, children’s length and weight were measured at childcare centers by the researchers. While anthropometric measurements were being taken, the children were barefoot and minimally clothed. Weight was measured with a pediatric scale (model SECA 354) and recorded to the nearest 100 g. Children’s length (12–24 months) was measured with the child lying down using an infant stadiometer placed on a flat, stable surface. If the child’s age was less than 2 years old and could stand but refused to lie down, height was measured and added 0.7 cm to convert it into length, according to international guidelines [22]. Height was always measured whether the child was two and more years old.

Body mass index (BMI) was computed as the body weight/height2 (kg/m2) ratio. Each child was classified according to the age- and sex-specific BMI (BMI for age) and BMI standard z-scores following the software Anthro-plus [WHO Anthro-plus software (https://www.who.int/childgrowth/software/en)].

### Socio-emotional development

Socio-emotional (SE) development was assessed with the Bayley Scales of Infant and Toddler Development – Third Edition (Bayley-III) [23], which is an adaptation of the Greenspan Social-Emotional Growth Chart [24]. Assessment of SE development occurs at the baseline and after the intervention. Bayley-III is designed for children aged between 1 to 42 months and used as a test of general neurodevelopment [3]. The socio-emotional scale identifies six stages (with substages), with milestones according to the child’s age, and measures behaviors associated with major milestones in functional and emotional development [23]. This is a comprehensive assessment completed by the child’s parent or caregiver, aimed at identifying and evaluating the child’s emotional competencies, including self-regulation, curiosity about the world, effective communication of needs, establishment of relationships, intentional utilization of emotions, and the use of emotional cues to solve problems. Percentiles of socio-emotional development were computed from the raw score and according to the criteria from the original scale [23]. The Cronbach’s alpha of the socio-emotional questionnaire from the original study is 0.90, which indicates a strong internal consistency [23].

### Intervention program

The intervention program was co-developed with important stakeholders, such as parents, health professionals, teachers/educators, and bloggers. Two sessions of focus groups with these important authors were carried out in person at the beginning of the trial (March 2019), emerging important topics such as nutrition and movement. In this context, the intervention program addressed topics such as, diet, sleep, physical activity, sedentary behavior, emotional self-regulation and children’s healthy everyday life in the family.

Six childcare centers received the intervention program (a total of 18 childcare teachers, 17 females), conducted between 29th January and 29th April 2020. Due to pandemic constraints, the sessions occurred in a mixed format, three in-person and five in an online format. The online sessions take place through the Colibri-Zoom® platform. Online sessions proceeded as expected, featuring the presentation of topics for each session, activities, and discussions of content, as initially planned in the in-person format. The 3-month training program had a total of 25 h.

The intervention program was approved by the Minister of Education, Scientific-Pedagogic Council for In-service Training (Conselho Científico-Pedagógico da Formação Contínua, Ministério da Educação), in the form of a training workshop.

The intervention program was based on the health promotion model of Nola Pender, which is one of the widely used models to promote healthy behaviors and control unhealthy ones. It is based on social cognitive theory and has core concepts: health promotion; health protection; individual characteristics and experiences; cognition associated with behavior, and health outcomes. The components of Penders’ theory provide a rich source of interventional strategies [25] and support the methodology adopted in the intervention program (i.e. participatory approach).

The intervention program followed two pathways: childcare teachers’ training provided by the research team and the intervention to children (in the classroom or to the family during the childcare closures) provided by the trained childcare teachers. To achieve the goals of the intervention program, each session was drawn to empower educators with creative activities and strategies focused on improving children’s health. The main objective was to empower educators with health promotion strategies and activities for implementation at childcare centers and in children’s homes by families. Some thematic experiences were suggested by the research team. Additionally, the childcare teacher contributed other experiences based on their observations following the implementation with the children. All the children from the intervention group (139 children) had contact with a trained childcare teacher.

The control group didn’t receive any intervention from the research team besides standard education and care. Childcare centers in this group were requested not to start any new health promotion activity initiatives during the 3-month intervention period.

### Statistical analysis

Central tendency measures and dispersion were used to obtain descriptive statistics according to the type of variables. Generalized linear models were conducted to examine the associations between socio-emotional status after intervention (outcome) according to the group of schools (i.e., control and intervention). Potential confounders included socio-emotional status before the intervention, demographic factors (e.g., sex, age), and socioeconomic status (mothers’ education).

The sample size was estimated considering the primary outcome of cognitive development as the variable of interest. In previous studies, cognitive development’s average (SD) was 93.3(8.0). To detect a 3% difference in cognitive development between groups (increasing, on average, the score of the cognitive development in the intervention group to 96.0), with errors type I and type II of 5% and 20%, respectively, we should have an effect magnitude of 0.35 and a final sample of 204 (102 for each group). As we randomized at the childcare center level, we considered the effect of design (variance inflation factor) given by the formula 1 + (m-1)*ICC, where m is the cluster size, and ICC is the intra-cluster correlation [26]. Based on previous studies, we considered an ICC of 0.01 [27], obtaining a design effect of 1.17. Knowing that in the city of Braga (where the study occurred), the number of children aged 12 months in each childcare center (cluster) is about 18, we should have a final follow-up sample of 204 children. However, we adjusted our sample size to the potential loss of subjects over the study period, which is usually < 25% [28]. Therefore, our final baseline sample should be 300 children belonging to 16 childcare centers (150 children and eight childcare centers per group).

The level of significance was established at 0.05. The data analysis was performed using IBM SPSS, version 27.0.

### Ethics approval and consent to participate

The study was approved by the Ethics Subcommittee for Life and Health Sciences of the University of Minho (CE.CVS 133/2018), and all the participants (children’s parents or caregivers) signed the informed consent. At the moment of evaluation, children assent to participate in the procedures. All methods were carried out in accordance with the declaration of Helsinki.

This cluster randomized controlled trial was registered on 09/09/2019 in the Clinical Trials database/platform (number NCT04082247), and CONSORT reporting guidelines were used [29].

## Results

Participants’ characteristics of the study, at baseline and follow-up, including sex, mean age, socio-emotional development, BMI z-score, and mothers’ education, are presented in Table 1.

**Table 1:** Characteristics of the sample at baseline and follow-up

	Baseline (*n* = 344)			Follow-up (*n* = 150)		
	Intervention (*n* = 139)	Control (*n* = 205)	*p*	Intervention (*n* = 62)	Control (*n* = 88)	*p*
Sex^a^			0.393			0.521
Male	64 (38.1)	104 (61.9)		27 (38.6)	43 (61.4)	
Female	75 (42.6)	101 (57.4)		35 (43.8)	45 (56.3)	
Age (months)	23.19 (6.1)	23.95 (6.4)	0.274	30.52 (6.6)	31.86 (6.2)	0.205
Socio-emotional development	56.79 (30.1)	55.10 (29.5)	0.663	59.54 (34.3)	54.57 (31.7)	0.444
BMI z-score	0.70 (1.0)	0.69 (1.0)	0.931	--	--	--
Mothers’ education^a^			0.079			0.200
Low education	56 (47.9)	61 (52.1)		27 (46.6)	31 (53.4)	
High education	74 (37.8)	122 (62.2)		57 (64.0)	32 (36.0)	

^a^Categorical variables are expressed as n(%); *p*-value from the chi-square test

In specific subgroup analysis (refer to Tables 2 and 3), a significant interaction was observed between mothers’ education level and groups of childcare centers (control and intervention), even with different levels of adjustment. According to the results, children who received the intervention and whose mothers have a low education level exhibited significantly higher socio-emotional development than the control group (B = 19.8; CI: 1.2, 38.5), even in the fully adjusted model (Model 3, B = 19.0; CI: 2.1, 35.9). Despite the increase in the socio-emotional percentile, even in the control group, the results are only significant in the intervention group.

**Table 2:** Subgroup analysis

	**Intervention**		**Control**	
	Baseline	Follow-up	Baseline	Follow-up
	Mean(SE)	Mean(SE)	Mean(SE)	Mean(SE)
**Socioemotional**				
**Mothers education**				
Low	52.2(4.5)	66.8(7.1)	42.6(4.0)	47.0(6.6)
High	59.4(4.5)	53.0(8.0)	61.4(2.6)	58.7(4.5)

**Table 3:** Subgroup analysis

	**Unadjusted**		**Model 1**		**Model 2**		**Model 3**	
	B (CI 95%)	*p*	B (CI 95%)	*p*	B (CI 95%)	*p*	B (CI 95%)	*p*
**Socioemotional**								
**Mothers education**								
Interaction		0.046		0.033		0.016		0.010
**Low**	19.8 (1.2; 38.5)	0.038	16.2 (16.1; 51.8)	0.083	21.5 (2.7; 40.2)	0.025	19.0 (2.1; 35.9)	0.028
**High**	-5.7 (-22.3; 10.8)	0.498	-11.8 (-26.3; 2.7)	0.112	-12.6 (-27.1; 2.0)	0.090	-12.8 (-27.6; 1.9)	0.089

Unadjusted model – Model with groups of childcare centers, mothers’ education and the interaction between mothers’ education and the group of childcare centers; Model 1 – adjusted for baseline socio-emotional development, groups of childcare centers, mothers’ education and the interaction between mothers’ education and the group of childcare centers; Model 2 – Model 1 plus adjustment for BMI z-score; Model 3 – Model 2 plus adjustment for sex and age

**Fig. 2:**
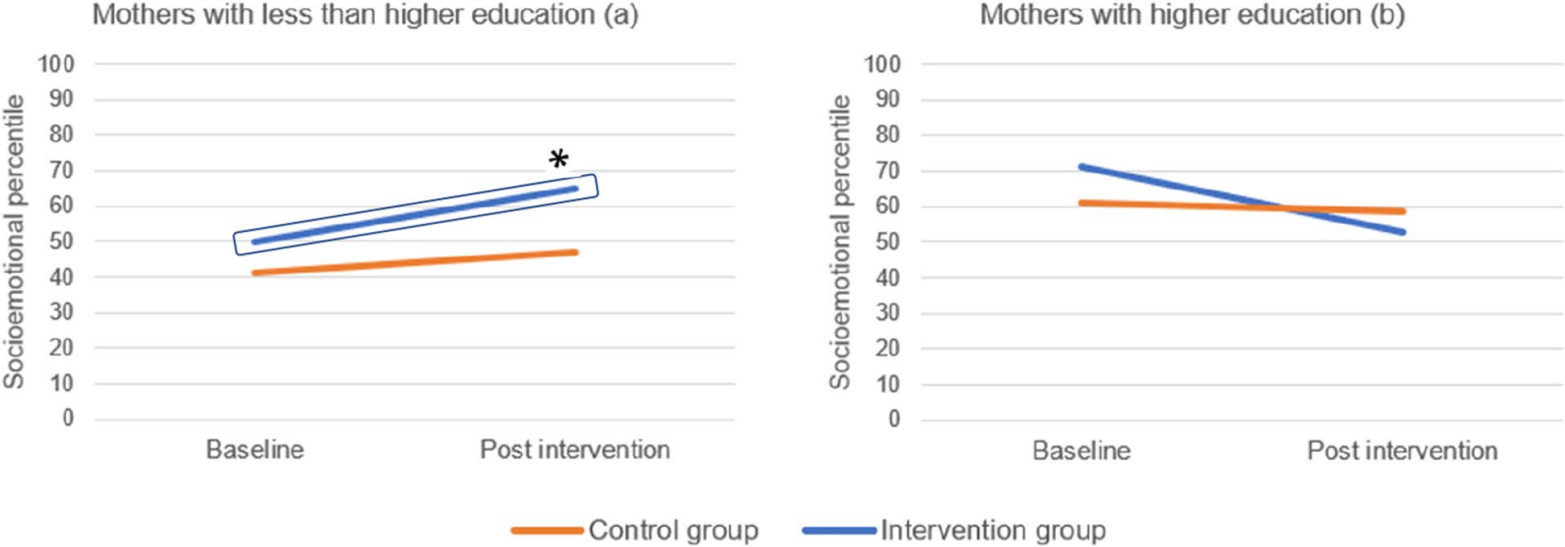
Socio-emotional percentile of children according to mothers’ education level at baseline and post-intervention. Legend: Socio-emotional percentile of children whose mothers have less than higher education (**a**) and socio-emotional development in children whose mothers have a higher education level (**b**) at baseline and post-intervention evaluation moments. The increase in the socio-emotional percentile was significantly higher in the intervention group

Figure 2 illustrates the tendency of socio-emotional development from baseline to after the intervention in both groups (e.g., control and intervention) and according to mothers’ education. In mothers with low education, intervened children had a significantly higher increase in their socio-emotional development than controllers.

## Discussion

In this study, we observed that children aged 12 to 42 months, whose mothers had lower levels of education appeared to derive greater benefits from the health promotion intervention program in terms of socio-emotional development. This discovery aligns with existing evidence and underscores the significance of empowering childcare teachers to foster the healthy development of all toddlers, especially those from families with lower socioeconomic status [20]. It is crucial to acknowledge the bidirectional relationship between socio-emotional development and health. For instance, socio-emotional development has been linked to various health indicators [30]. Stress or negative events have been correlated with socio-emotional disorders or adoption of coping strategies, potentially leading to health problems [2]. Socio-emotional factors can directly impact eating behaviors and hunger sensations, consequently influencing overall health outcomes. Previous studies have indicated a significant association between socio-emotional status and hunger sensation in children and adolescents, with those experiencing more negative emotions reporting elevated levels of hunger [30, 31]. Additionally, emotional eating, or consuming food as a coping mechanism for negative emotions, has been significantly correlated with adverse health outcomes, including obesity and metabolic syndrome [2].

Intervention programs involving childcare teachers and parents have proven effective in promoting children’s health [32, 33]. Parents and family members play a crucial role in shaping children’s health habits and choices, especially during the early years of life.

Children glean behaviors and attitudes toward healthy lifestyle practices, such as consuming nutritious foods, engaging in physical activity, and ensuring adequate sleep, from their parents [2, 34]. Simultaneously, childcare teachers wield significant influence in promoting children’s development [35], given their responsibility for children’s care and learning during the day. Research indicates the stable and secure relationships with caregivers, including childcare teachers, are pivotal for fostering socio-emotional development in young children [4]. Furthermore, parental school involvement and the development of socio-emotional skills have emerged as predictors of various outcomes in young adulthood, encompassing educational attainment, health-compromising behavior, economic well-being, and mental health [36]. Not surprisingly, children whose mothers have higher education levels maintained their socio-emotional development, despite facing challenges related to Covid-19 constraints. Several external factors associated with pandemic restrictions may have contributed to this outcome. The intervention program was implemented, and post-intervention evaluations were conducted in Portugal during a period of confinement measure. Mothers with higher education levels likely adapted to working from home during successive lockdowns, balancing work responsibilities, safeguarding their family’s health, and supporting their children’s needs and education [37]. Many of these mothers could have more than one child, each with varying ages and developmental levels. It was a challenging period, marked by heightened stress and parental anxiety. Although not variables assessed in our study, according to other research, these factors could potentially exert a negative influence on the socio-emotional development of children whose mothers experience an excessive workload [38–40]. Furthermore, mothers with higher education levels are frequentlymore involved in their children’s development [20, 21, 34]. Consequently, additional studies are necessary to elucidate the influence of maternal education, especially in emergency situations, on children’s development during these crucial stages. There is also the possibility that children augmented their screen time during the intervention program implementation [41], a factor that may have adverse effects on socio-emotional development [41–43]. This study exhibited several strengths. Its longitudinal design enables the evaluation of the temporal sequence in socio-emotional development. Furthermore, socio-emotional development was appraised using a scale specifically tailored for children aged 1 to 42 months, recognized as a comprehensive measure of overall development [3, 23]. The assessments were conducted by a specialized research team at childcare centers during the baseline evaluation.

For data analysis, generalized linear models were employed, with controls for potential confounding factors associated with socio-emotional development in children, such as demographic variables (e.g., sex, age), body mass index (z-score) [3, 44], and mothers’ education level [20]. Furthermore, it is noteworthy that the majority of existing studies have concentrated on socio-emotional development in children aged three and above. For example, Svandová et al. [45] investigated the divergence between cognitive and socio-emotional development among children born with low birth weight at ages 5 and 9, while Valero-García et al. [46] explored the impact of both parents’ use of behavioral regulation with food and children’s emotional self-regulation (with and without overweight or obesity) at ages 4 and 7.

With regard to intervention programs, studies have explored the influence of such programs on health-related behaviors and outcomes in children across various age groups, although a majority have been implemented at the preschool or school level. For instance, Rosário et al. [32] demonstrated the effectiveness of a health promotion intervention program in increasing fruit consumption as a dessert duringlunch and dinner among children aged 6 to 12 years. Similarly, Wang et al. [47] investigated the effectiveness of a school-based intervention program in reducing obesity and improving physical fitness levels related to hypertension in children with intellectual disabilities. However, to the best of our knowledge, no prior studies have investigated the impact of an intervention program targeting childcare teachers/parents on the socio-emotional development of children under 3 years old.

As anticipated, this study experienced some participant dropouts, primarily attributed to pandemic constraints. Nevertheless, the disparity between the children in both groups (control and intervention) who withdrew from the study was not statistically significant compared to those who remained, suggesting that the dropouts did not significantly compromise the study’s internal validity. The characteristics and outcomes of the toddlers who dropped out were likely comparable to those who completed the study, mitigating potential bias in the results.

This study is subject to certain limitations, primarily stemming from pandemic-related restrictions. Firstly, the originally planned in-person format of the intervention program had to be modified into an online format. Despite successful enrollment all participants in the new format, direct observation of their engagement with the program was not feasible.

Nevertheless, the research team continued to stay in touch with participants even after the conclusion of the intervention program, offering additional support during challenging periods. Second, the follow-up assessments were carried out online, necessitating reliance on self-reported information from parents, including anthropometric measures. This could potentially introduce some bias or measurement error into the data. Furthermore, the study lacked a long-term follow-up assessment, which would have offered valuable insights into the sustainability of the intervention program’s effects on socio-emotional development.

At the analysis level, we acknowledge a limitation in exclusively considering the mother’s education rather than both parents’, a factor that could have added more precision to the data.

Our findings underscore the critical importance of promoting socio-emotional development in toddlers, aligning with existence evidence. Families facing socioeconomic vulnerabilities may particularly need additional support and attention from the research community, potentially benefiting from supplementary resources and interventions.

The present study underscores the effectiveness of developing intervention programs targeted towards childcare educators as a successful strategy for promoting socio-emotional development in toddlers. While limited have specifically implemented such programs for children aged less than 3 years, our findings demonstrate the potential impact of interventions targeting childcare teachers/parents on positively influencing children’s socio-emotional development. By equipping these caregivers with the necessary knowledge and skills, interventions can not only enhance children’s socio-emotional outcomes but also cultivate a supportive and nurturing environment for their growth and development. Furthermore, such interventions have the potential to address the disparities in socio-emotional development observed in children from socioeconomically vulnerable families, who may derive significant benefits from this type of intervention. Consequently, our study emphasizes the importance of future research continuing to explore the effectiveness of these interventions in promoting toddlers’ socio-emotional development. These findings offer valuable insights for the design of future intervention programs aimed at improving children’s socio-emotional development.

## Data Availability

The datasets used and/or analyzed during the current study are available from the corresponding author upon reasonable request.
